# RNA Dependent RNA Polymerases: Insights from Structure, Function and Evolution

**DOI:** 10.3390/v10020076

**Published:** 2018-02-10

**Authors:** Sangita Venkataraman, Burra V L S Prasad, Ramasamy Selvarajan

**Affiliations:** 1 Department of Biotechnology, Acharya Nagarjuna University, Nagarjuna Nagar, Guntur 522510, India; 2 Amity Institute of Biotechnology, Amity University Haryana, Manesar, Gurgaon 122413, India; dr.prasad.bvls@gmail.com; 3 ICAR National Research Centre for Banana, Thayanur Post, Tiruchirapalli 620102, India

**Keywords:** RNA viruses, RNA-dependent RNA polymerase, domains, motifs, structure-based Phylogeny, inhibitor complexes

## Abstract

RNA dependent RNA polymerase (RdRp) is one of the most versatile enzymes of RNA viruses that is indispensable for replicating the genome as well as for carrying out transcription. The core structural features of RdRps are conserved, despite the divergence in their sequences. The structure of RdRp resembles that of a cupped right hand and consists of fingers, palm and thumb subdomains. The catalysis involves the participation of conserved aspartates and divalent metal ions. Complexes of RdRps with substrates, inhibitors and metal ions provide a comprehensive view of their functional mechanism and offer valuable insights regarding the development of antivirals. In this article, we provide an overview of the structural aspects of RdRps and their complexes from the Group III, IV and V viruses and their structure-based phylogeny.

## 1. Introduction

RNA genomes dominate the world of viruses. Their success results from the possibility of accommodating rapid changes via mutations, which aids in countering constant challenges imposed by the host physiology [1]. Of the many factors that substantiate the role of RNA dependent RNA polymerases (RdRps) in viral evolution, the primary one concerns high rate of error during the copying (≈10^−4^) process due to the lack of a proofreading exonuclease activity [1]. The increased rates of mutation in the progeny viral population allows some of the variants to be selected under the pressures imposed by the host defense mechanisms and other environmental factors [2]. In addition, the process of strand switching by RdRps during replication allows for recombination that facilitates rearrangement of genes or acquisition of new ones from other viruses or hosts [3].

RdRps are multi-domain (α and β) proteins belonging to Structural Classification of Proteins (SCOP) class 2.7.7.48. They catalyze RNA-template dependent formation of phosphodiester bonds between ribonucleotides in the presence of divalent metal ions [4]. The initiation of synthesis occurs at the 3’-end of the template in a primer-dependent or independent manner and proceeds in the 5′ → 3′ direction. The average length of the core RdRp domain is less than 500 amino acids and is folded into three subdomains, viz., thumb, palm, and fingers resembling a right-handed cup [4]. The active sites of RdRps from different RNA viruses are conserved and show resemblances to those of other enzymes such as reverse transcriptases and DNA polymerases indicating their similar role in nucleotidyl transfer reactions [5,6,7,8].

Many viral polymerases possess additional domains such as methyltransferase or endonuclease domain to carry out functions associated with RNA synthesis. The polymerase domain may also interact with other host factors for efficient polymerization and to discriminate activities such as genome replication and mRNA transcription [3,9,10,11]. The host factors include translation factors, protein chaperones, RNA-modifying enzymes, and a few other cellular proteins. These together with the RdRps, constitute the viral replication complexes (VRCs) [3,9]. The VRCs differ in their composition, subcellular location, and interaction with the viral RNA templates. In many Group IV viruses, VRCs associate with the host membranes to protect the viral RNAs and help segregation of products and templates during replication [12,13].

In the current review, X-ray crystal structures of RdRps (available as of September 2017) belonging to PROSITE [14] accession no. PRU00539 were procured from the Protein Data Bank (PDB) [15] (Table 1) and analyzed for their unique and shared features. These include the RdRps of double-stranded (ds) RNA viruses from Group III, single-stranded (ss) positive sense (+) RNA viruses from group IV infecting Eukaryotes, Bacteriophages from Group III and Group IV, and ss negative sense (−) RNA viruses from Group V with segmented or unsegmented genomes (Table 1). Comparison of the RdRp structures in association with different ligands and inhibitors helped in a comprehensive understanding of the mechanism of action and potential strategies for the design of antivirals. The structure-based phylogenetic analysis provided deeper insights into the evolutionary connections between disparate families of viruses. The figures in the manuscript were generated using Chimera [16], PyMOL [17] and Inkscape.

## 2. Structural Features

### 2.1. The Subdomains

**Figure 1:**
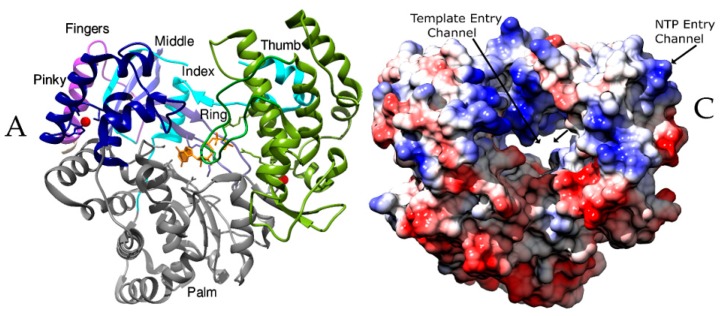

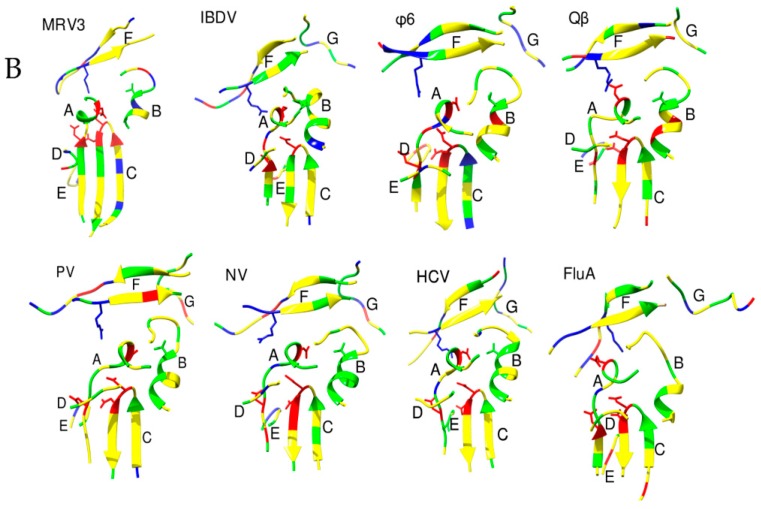
The architecture of RdRp. Panels A and C show the RdRps in the same orientation, viewing down the active site cleft. (**A**) RdRp of *Japanese encephalitis virus* (JEV) (PDB ID: 4mtp) [32] showing the thumb (Green), palm (Grey) and fingers subdomains (Blue). The linker connecting the RdRp and methyltransferase domains is depicted in purple. The components of fingers subdomains including Index (Cyan), Middle (Blue), Ring (Cornflower blue) and Pinky (Navy Blue) are indicated. The metal ions (Zn) are shown as spheres of red and a GTP that is bound at the active site is shown as sticks in orange color. The characteristic priming loop of *Flaviviridae* is shown in olive green that fills up the active site and facilitates de novo initiation. (**B**) The structural motifs A to F of RdRps from representative viruses of families: *Reoviridae* (Simian rotavirus SA11 (SiRV)), *Birnaviridae* (*Infectious bursal disease virus* (IBDV)), *Cystoviridae* (*Pseudomonas phage φ6* (φ6)), *Orthomyxoviridae* (*Influenza A virus* (FluA)), *Picornaviridae* (Poliovirus type I (PV)), *Caliciviridae* (Human Norovirus (hNoV)), *Flaviviridae* (*Hepatitis C virus* (HCV)), and *Leviviridae* (*Bacteriophage Qβ* (Qβ)) are shown in ribbon representation. The polar residues are presented in green color, acidic in red and basic in blue. The conserved aspartates of motif A, threonine of motif B, and arginine of motif F are represented as sticks. (**C**) The RdRp of Coxsackievirus B3 (CVB3, PDB ID: 4zpc) [47] from *Picornaviridae*, shown in surface representation and colored based on electrostatic potential, reveals the NTP entry channel and the template channel. The channels are lined with positively charged residues and promote the binding of the template RNA, the primer, and NTPs for catalysis.

The core RdRp domain consists of the thumb, palm and the fingers subdomains that are primarily involved in template binding, polymerization, nucleoside triphosphate (NTP) entry and associated functions (Figure 1A). The palm subdomain is at the junction of the fingers and the thumb subdomains and houses most of the structurally conserved elements involved in catalysis. The catalytic aspartates and the RNA Recognizing Motif (RRM) comprising three β-strands are present in the palm subdomain [5]. The subdomain selects NTPs over deoxy NTPs and catalyzes the phosphoryl transfer reaction by coordinating two metal ions [5,6,18]. The palm subdomain of the RdRps of Picornaviruses and Caliciviruses are conformationally dynamic, as they undergo significant restructuring following NTP binding to facilitate catalysis [19,20]. The catalytic motifs of Birnaviral palm subdomain shuffle in sequence without the change in the overall 3D structure leading to an unusual topology [21,22].

The thumb subdomain harbors residues that are involved in packing against the template RNA and stabilizing the initiating NTPs on the template [6]. This subdomain also facilitates the translocation of the template following polymerization by accommodating large conformational rearrangements [6]. In viruses that do not separate the template and product strands, a structurally conserved helix of the thumb subdomain makes significant interactions with the minor grove of the RNA duplex [23]. The thumb subdomain is the most diverse among the available RdRps and differs in size and complexity based on the mode of initiation of replication. RdRps from *Picornaviridae* and *Caliciviridae* that use a primer to initiate replication have small thumb subdomains to facilitate the binding of primer such as viral protein genome-linked (VPg), while RdRps from *Flaviviridae* have large thumb subdomains to aid de novo initiation of replication [19,20,24,25]. RdRps of families such as *Flaviviridae*, *Leviviridae*, *Orthomyxoviridae*, and *Reoviridae* with de novo initiation often possess a “priming loop” that emanates from the thumb subdomain and acts like a stabilizing platform during initiation [26,27,28,29]. Additionally, there is a unique C-terminus that folds back into the active site cleft regulating RNA synthesis [25,30].

The fingers subdomain plays a significant role in setting the geometry of the active site. It serves to hold the template RNA in place and facilitates polymerization [19]. The fingers subdomain interacts with the major groove of the template RNA and aids in recognition and binding. This subdomain is composed of intertwined fingers (Figure 1A), viz. Index, Middle, Ring, and Pinky [31,32], that extend in many viruses as β strands (“fingertips”) and establish contact with the thumb subdomain closing the active site [6]. The fingertips comprise of a set of three β strands in *Bovine viral diarrhea virus* (BVDV) [33,34], four each in *Hepatitis C virus* (HCV) [35,36,37] and Caliciviruses [38,39,40,41], and six in *Pseudomonas phage φ6* (φ6) [42,43] and *Pseudomonas phage φ12* (φ12) [44]. A twisted, four-stranded β-sheet forms the fingertip regions of *Birnaviridae* [22,45] and *Picobirnaviridae* [46]. Surprisingly, these do not interact directly with the thumb subdomain [21,22,45,46].

### 2.2. The Motifs

A set of seven structural motifs, A to G, characterize the conserved structural component of RdRps indicating their pivotal role in catalysis [5,48]. The motifs retain remarkable similarity in their structure and disposition across various groups of viruses (Figure 1B). Motifs A to E are housed in the palm subdomain while motifs G and F are part of the fingers subdomain. The thumb subdomain for ss (+) RNA viruses and ds RNA viruses possess an additional motif H [18]. This motif is associated with the fingers subdomains for the segmented ss (−) RNA viruses as a single β strand and stabilizes motif B [45,46].

Motif A, formed by a β-strand, continues either as a helix or a loop into the fingers subdomain [48]. It houses the catalytic motif DX_2-4_D in which the first aspartate is invariant in various RdRps. The second aspartate together with a conserved asparagine from motif B plays a crucial role in the discrimination of NTPs over deoxy NTPs by forming a hydrogen bond with 2′OH of the incoming NTP [6]. A conserved lysine replaces the second aspartate in RdRps of ss (−) RNA viruses allowing them to use manganese instead of magnesium as the cofactor [49,50,51]. The positioning of motifs A and C in the RRM of Picornaviruses is off-base until the binding of the correct NTP, whereupon they are realigned [19].

Motif B of the palm subdomain assists the binding of the template RNA and in substrate discrimination [52]. It is mainly a loop that connects a β strand of the fingers subdomain to the N-terminal helix arising from the palm. Being flexible, it serves as a hinge to accommodate critical conformational changes associated with template and substrate binding [52]. A conserved glycine occurs at the junction of the loop and the helix and is indispensable for the polymerase function [53]. Motif B houses a threonine within the α-helix facing the active site which is conserved in most Group IV and dsRNA viruses but is absent in the Group V viruses [52].

One of the most conserved motifs, Motif C, is formed by a loop and two flanking β-strands. The loop houses the conserved GDD motif essential for binding the metal ions [5,18,54,55]. Motifs A and C are spatially juxtaposed and contribute to the RRM. The conserved aspartates from GDD and the first aspartate from DX_2-4_D align at the tip of the RRM aiding in efficient catalysis (Figure 1B). The GDD motif is lacking in Birnaviral RdRps [21,22,45] that results in lowered efficiency of the polymerases. In segmented ss (−) RNA viruses, the glycine of GDD motif is replaced by a serine [56].

Motif D, comprising of an alpha helix and a flexible loop, lies adjacent to the β sheet of the palm subdomain [48,57]. It serves as a pivot for conformational changes associated with correct NTP binding because of a conserved glycine (Figure 1B) [58]. NMR studies have indicated the inability of motif D to achieve its optimal conformation for catalysis when an incorrect nucleotide is incorporated, thereby demonstrating its role in the selection of NTPs [57]. Motif D is one of the most dynamic elements as its position is observed to vary by as much as 6 Å in different RdRp structures [57]. It is known to enable the movement of the thumb subdomain during elongation [59,60]. A conserved lysine in this motif serves as a general acid in the crucial function of deprotonation of the pyrophosphate leaving group [59].

Motif E is a β-hairpin located at the junction of the palm and the thumb subdomain. It is also termed as “the primer grip” as it aids the correct positioning of the 3′ hydroxyl group of the primer for catalysis [48,61]. An aromatic residue that lies at the N-terminal side facing motif C is one of the most conserved aspects of this motif that appears to be absent in *Bacteriophage Qβ* (Qβ) [18,28].

Motif F, formed by a loop and a β-strand, interacts with the phosphate group of incoming NTP. It comprises of positively charged residues that shield the negative charges of the phosphate groups of incoming NTPs by lying directly over the palm subdomain [6,48]. The C-terminal region of motif F houses a conserved arginine. In La crosse virus (LACV) [62] and Influenza viruses, motif F is ordered upon the binding of 5′ viral RNA [56,63]. In Picornaviruses, positively charged residues of motif F participate in the uridylylation of VPg [19] while in Flaviviruses, it is proposed to promote RNA synthesis [30].

Motif G comprises a loop that is a part of the template entrance tunnel in ss (+) RNA viruses [19,20] and fingers subdomain in dsRNA viruses [22,29,45,64,65]. In the segmented ss (−) RNA viruses, motif G is composed of a helix that interacts with the priming NTPs [56,62,66]. It is a component of the polymerase acidic (PA) subunit of *Influenza A virus* (FluA) [49,67] and *Influenza B virus* (FluB) [50], Polymerase 3 (P3) subunit of *Influenza C virus* (FluC) [51] and PA C-terminal (PA-C) like domain of LACV [62,63].

### 2.3. The Channels

The RdRps have channels or tunnels that traverse in different directions connecting the catalytic centers with the exterior [7,48]. These channels have emerged as important targets for the development of drugs. The entry channels are lined with positively charged residues and hence, favor the entry of NTPs and the template RNA into the active site [6,68]. The nucleotide entry channel facilitates the entry of the substrate and divalent cations into the central active site cavity (Figure 1C). This channel is known to participate in the release of the pyrophosphate moiety after polymerization [69]. The template channel is involved in template recognition and driving the NTPs towards the catalytic center (Figure 1C) [68,70]. Motif G of the conserved structural motifs lines the entry of the template channel while motif B is shown to form the base [18,19]. In *Reoviridae*, this channel is formed by the residues of the N-terminal domain along with the fingers and thumb subdomains. It binds the 3′ end of (−) RNA during transcription and (+) RNA during genome replication [69,71]. In Qβ replicase complex, the plasticity of template channel was proposed to be essential for the binding of legitimate template RNA during initiation of replication [72]. The Picornaviral RdRps show significant flexibility in their template channels and hence, are potential targets for the development of antivirals [68]. The template channel showed possibilities for inhibitor design in pathogenic viruses from *Flaviviridae* such as JEV [31], *Dengue virus* (DENV) [73,74,75,76], *Zika virus* (ZIKV) [77,78,79], *West nile virus* (WNV) [80] and HCV [35,81,82]. These viruses possess a narrow template channel that can accommodate only ss RNA and NTPs owing to larger thumb subdomains [24,25,30]. In contrast, *Picornaviridae* and *Caliciviridae* have a wider template channel and accommodate both a template and a protein primer [19,20].

The RNA exit channel is formed by both the palm and the thumb subdomains and serves as the exit path for the template, as well as, newly synthesized RNA. In *Reoviridae*, this channel is the largest of all, extending through the C-terminal domain [69]. In the Rotaviral VP1, an α-helical plug of unknown function extends 15 Å into this exit channel reducing its diameter [71]. A unique fourth channel in *Reoviridae* serves as the exit channel for the (+) RNA products of transcription [69]. However, the RdRps of *Cystoviridae* [42,44,83], *Birnaviridae* [21,22], *Picobirnaviridae* [46] and the Group IV viruses possess only the regular channels, one each for NTP and metal ion entry, template entry and the exit of the template and product RNA [23,38,39,40,41,84,85,86,87,88,89,90]. Along with these channels, the influenza viruses possess a fourth channel dedicated exclusively for the exit of template RNA [49,50,51]. The template exit channel lies on the same side as the template entry channel. The Large polymerase protein (L) of Machupo virus (MACV), a segmented virus of the *Arenaviridae* family [91], *Human picobirnavirus* (hPBV) [46] and *Vesicular stomatitis virus* (VSV) [92] is proposed to have the four-channeled architecture similar to *Reoviridae* [69].

### 2.4. Additional Structural Elements

**Figure 2:**
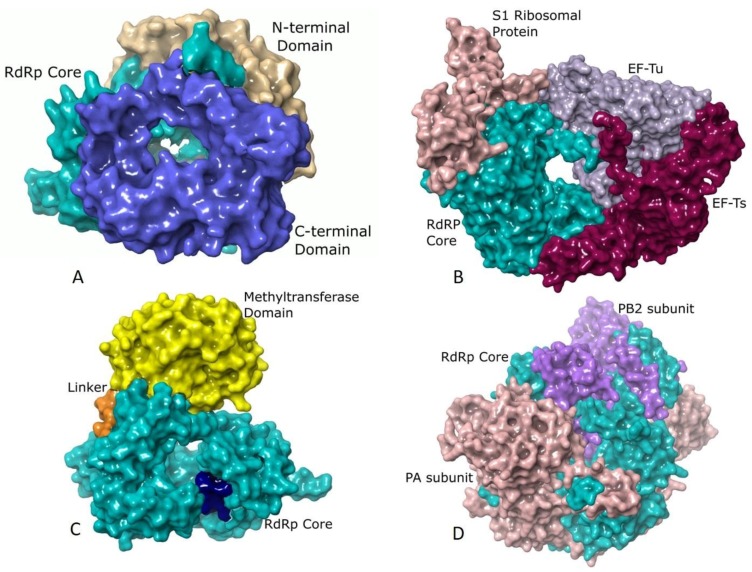
Additional structural elements of different RdRps: (**A**) The surface representation of RdRp of Mammalian orthoreovirus 3 (MRV3) (PDB ID: 1n35) [29] showing the core (residues 371–890, colored turquoise) in association with the C-terminal bracelet domain (shown in blue) composed of residues 891–1267 and the N-terminal domain (depicted in tan) spanning residues 1–370. While the N-terminal domain restricts the movements of the thumb and fingers subdomains, the C-terminal domain resembles the sliding clamp of DNA polymerases in structure and has a positively charged opening of 20 Å diameter. (**B**) Qβ polymerase (PDB ID: 4r71) [72] shown in surface representation with the core RdRp (β-subunit, Chain B, residues 1–571) colored turquoise, host translation elongation factors EF-Tu (Chain A, residues 2–1393, colored mauve) and EF-Ts (Chain C, residues 4–1394, colored dark magenta) and the S1 ribosomal protein (Chain E, residues 1–171, colored rosy brown). The extensive interactions of EF-Tu with the β-subunit is believed to aid in rapid separation of the duplex RNA that is formed during polymerization thereby allowing exponential amplification of phage genome. The S1 protein is involved in recognizing (+) strand of Qβ [72]. (**C**) The RdRp of *Dengue virus* (DENV) (PDB ID: 4hhj) [73] rendered in surface showing the core (residues 272–900, colored turquoise) with the priming loop residues 789–805 shown in dark blue, the methyltransferase domain (residues 1–262) in yellow and the linker (residues 213–271) in orange. The methyltransferase domain is an essential part of the replication machinery that catalyzes 5’-RNA capping and methylation during viral genome replication. (**D**) The *Influenza B virus* (FluB) replicase (PDB ID: 4wrt) [50] consists of the PA (Chain A, residues 1–726, colored sandy brown), PB1 (Chain B, residues 1–752, colored turquoise) and PB2 (Chain C, residues 1–770, colored purple) domains are shown in surface representation. PB1 has polymerase activity, PB2 possesses a cap-binding domain and PA contains an endonuclease domain. The PA and PB2 domains lie towards the N- and C-terminal domains of PB1, respectively.

Although the core polymerase structure retains significant similarity in various groups, there are additional domains in individual viruses that support other processes accompanying RNA synthesis. λ3 polymerases of Reoviruses [29], Rotaviral VP1 [71], and RdRp of *Bombyx mori* Cytoplasmic polyhedrosis virus (BmCPV) [65] resemble large caged molecules surrounded by additional elements comprising the long N- and C-terminal extensions (Figure 2A). The N-terminal domain lies on one side of the active site cleft and wraps the continuous surface between the fingers and thumb subdomains. The C-terminal domain is an annular structure popularly referred to as the “bracelet domain” possessing a large opening capable of admitting dsRNA. Interactions of Rotaviral VP1 with the innermost capsid protein (VP2) helps in anchoring VP1 within the core and serves as a cofactor in stimulating the polymerase to initiate ds RNA synthesis using (+) RNA as a template (Figure 2A) [93]. The Birnaviruses [22,45] and Picobirnaviruses [46] lack the prominent N and C terminal domains. The latter shows a characteristic long C-terminal insertion loop that partially occupies the active site similar to the priming loop of Flaviviruses [30].

The replication machinery of bacteriophage Qβ from *Leviviridae* is a tetrameric protein complex comprising of the RdRp (β subunit) and three host factors that include the translational elongation factors, EF-Tu and EF-Ts and ribosomal protein S1 (Figure 2B) [28,72,94,95]. EF-Tu and EF-Ts are possibly involved in chaperone-like activities for the expression and assembly of the core Qβ replicase [94]. EF-Tu plays a significant role in the separation of the ds RNA of the template and the growing RNA at the elongation stage by forming an exit tunnel in association with the β-subunit [80]. The RdRps of phage φ6 [83] and φ12 [44] are similar to those of Reoviruses and possess extended N and C-terminal domains [29].

The structure of the core polymerase of Group IV viruses including the families of *Picornaviridae* [19], *Caliciviridae* [20], and *Flaviviridae* [30] exhibit extensive interactions between the fingers and thumb subdomains through the fingertip regions. These interactions constrain the movement of thumb subdomain relative to the fingers thereby restricting the conformational changes associated with the template and primer binding. The *Picornaviridae* [23,70,84,85,86,87,88,89,90] and *Caliciviridae* [38,39,40,41] core polymerases are small (≈50 kDa) lacking any additional domains. They have a large hollow near the active site to facilitate the binding of VPg, and this limits the size of the thumb subdomain. However, the Flaviviral polymerases have a large thumb subdomain as they use de novo initiation of replication [36,73,77,78,80]. They also have a methyltransferase domain fused at the N-terminus of the core polymerase that carries out capping and methylation of RNA cap using S-adenosyl methionine [96,97] (Figure 2C). The methyltransferase domain differs significantly in length and sequence between Flavi, Hepaci, and Pestiviruses within *Flaviviridae* [24,25,30].

In the segmented ss (−) RNA viruses from *Orthomyxoviridae*, a heterotrimeric RdRp binds the 5′ and 3′ regions of the genome forming a viral ribonucleoprotein complex (vRNP) [49,50,51]. The PA subunit lies towards the N-terminal end while the PB2 subunit is located towards the C-terminus of PB1 (Figure 2D). In Influenza viruses, the endonuclease activity of the N-terminal domain of the PA protein aids in cap-snatching. The PB1 subunit resembles a ring-like structure possessing the fingers, palm and thumb subdomains (Figure 2D). Despite low sequence similarities, the monomeric RdRps of *Bunyaviridae* share distinctive resemblances to the heterotrimeric RdRps of Influenza viruses [62,63]. Their single large (L) chain is folded into the PA, PB1, and PB2 like subdomains with a head-to-tail correspondence with Influenza viruses [62,63]. The non-segmented ss (−) RNA viruses have multifunctional, L polymerase proteins within the virions [84] that possess not only the RdRp activity but also catalyze mRNA capping reactions and polyadenylation of viral mRNAs. They harbor three domains viz. the RdRp, polyribonucleotidyl transferase (PRNTase), and methyltransferase domains [92]. The studies using VSV show that the core domain is like that of the Reoviruses except that it lacks the C-terminal bracelet domain [98]. The core, as well as the N-terminal domain of VSV, share structural features with PB1 and PA domains, respectively, of Influenza viruses [92,98]. The L proteins of Rabies, Ebola, Measles, and Respiratory syncytial viruses are homologous to that of VSV [92,99].

## 3. Structure-Based Phylogeny of RdRps

**Figure 3:**
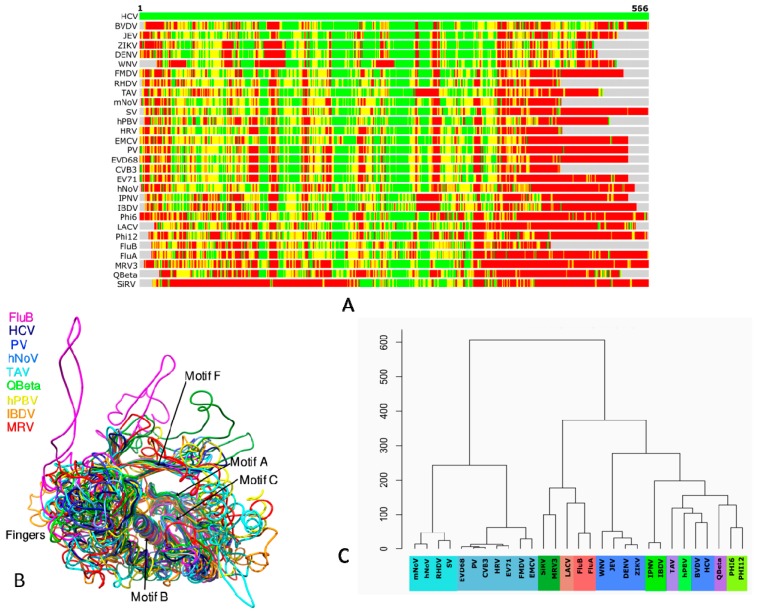
Structure-based alignment and phylogeny of representative RdRps listed in Table 1 (excluding VSV and BmCPV) using STRALCP server run with default parameters [89]: (**A**) Structural alignment of the RdRps of BVDV (PDB ID: 2cjq) [33,34], *West nile virus* (WNV) (PDB ID: 2hcn) [80], DENV (PDB ID: 4hhj) [73], *Zika virus* (ZIKV) (PDB ID: 5wz3) [78], JEV (PDB ID: 4k6m) [77], *Thosea asigna virus* (TAV) (PDB ID: 4xhi) [106], Coxsackievirus B3 (CVB3) (PDB ID: 4zpc) [70], Human rhinovirus 16 (HRV) (PDB ID: 1xr7) [86], PV (PDB ID: 1ra6) [23], *Foot-and-mouth disease virus* (FMDV) (PDB ID: 1u09) [87], Encephalomyocarditis virus 1 (EMCV) (PDB ID: 4nyz) [88], Enterovirus A71 (EV71) (PDB ID: 5f8n) [89], Enterovirus D68 (EVD68) (PDB ID: 5xe0) [90], Murine Norovirus (mNoV) (PDB ID: 3uqs) [38], hNoV (PDB ID: 4nrt) [39], *Sapporo virus* (SV) (PDB ID: 2uut) [40], *Rabbit hemorrhagic disease virus* (RHDV) (PDB ID: 1khw) [41], FluA (PDB ID: 5m3h) [49], FluB (PDB ID: 4wrt) [50], LACV (PDB ID: 5amq) [62], Qβ (PDB ID: 3mmp) [28], φ6 (PDB ID: 1hhs) [42], *Pseudomonas phage φ12* (φ12) (PDB ID: 4gzk) [44], MRV3 (PDB ID: 1n35) [29], SiRV (PDB ID: 2r7r) [71], IBDV (PDB ID: 2pus) [45], *Infectious pancreatic necrosis virus* (IPNV) (PDB ID: 2yi9) [22], and *Human picobirnavirus* (hPBV) (PDB ID: 5i61) [46] with HCV RdRp (PDB ID: 1nb4) [35]. The colored bars show Cα–Cα distances at each position from the amino-terminal (left) to the carboxy-terminal end (right) between HCV (top bar) and other structures. The colors indicate distances between aligned residues ranging from green (below 2 Å), yellow (below 4 Å), orange (below 6 Å), to red (above 6 Å). (**B**) Structural superposition of the core region of select viral RdRps is shown. The striking similarity of structural components around the catalytic center is evident. While the palm is seen to be the most conserved subdomain, significant differences are observable in the fingers and thumb subdomains of individual viruses. (**C**) Structure-based phylogeny of viral RdRps is presented. Groups of ss (+) RNA viruses are shown in shades of blue: pale blue indicating *Caliciviridae*, medium blue representing *Picornaviridae* and dark blue indicating *Flaviviridae*. dsRNA viruses are shown in shades of green: *Reoviridae* in dark green, *Birnaviridae* in medium green, *Picobirnaviridae* in light green, and *Cystoviridae* in lime green. *Permutotetraviridae* and *Leviviridae* from Group IV are presented in light and medium purple, respectively. ss (−) RNA viruses are colored in shades of Red, *Orthomyxoviridae* in dark red and *Bunyaviridae* in light red.

RdRp is the most conserved gene in RNA viruses that is ideally suited to understand their evolutionary patterns [100,101]. The molecular phylogeny of RdRps demonstrates diversity in hosts, capsid morphologies and genomic features arising out of the loss of ancestral genes, gene exchange between distant viruses and transfer of viruses between hosts [102]. Representative RdRps of different viruses (Table 1) from various groups determined (preferably in apo form) at high resolution were analyzed using STRALCP (http://proteinmodel.org/AS2TS/STRALCP/stralcp.cgi) for clustering protein domains based on their structural similarities (Figure 3) [103]. In this approach, global and local structural similarities between pairs of protein structures are used to identify spans of conserved regions and cluster them. The low resolution of the RdRp structures of FluC (3.9 Å) [51], VSV (3.8 Å) [92], and BmCPV (3.9 Å) [65] necessitated their exclusion from the analysis. Figure 3A shows the structural alignment of HCV with the rest of the PDBs with green, yellow, orange and red colors representing RMSDs below 2 Å, 4 Å, 6 Å and above 6 Å, respectively. While the RMSDs of N and C-terminals are significantly higher in the alignment, the regions in the center corresponding to the core catalytic segments are structurally conserved (Figure 3A,B).

The structure-based phylogenetic tree shows two major clades (Figure 3C). The first comprises *Caliciviridae* and *Picornaviridae*, and the second clade includes *Flaviviridae* and other families from Groups III and V. Indeed, *Flaviviridae* is evolutionarily distant from both *Picornaviridae* and *Caliciviriadae* though they possess a similar type of genome. It is instead closer to viruses from Groups III and V. This may be due to the possession of structural features necessary for accommodating primer-independent initiation for replication necessitating a large thumb subdomain and a priming loop [33,35,73,77,78,80]. The second clade is further divided into two sub-clades: the first comprising *Orthomyxoviridae*, *Bunyaviridae*, and *Reoviridae*, and the other including the rest of the viruses from *Flaviviridae*, *Permutetraviridae*, *Birnaviridae*, *Picobirnaviridae*, *Cystoviridae*, and *Leviviridae*. The *Flaviviridae* segregate from the ancestral node into two groups, one comprising of the Flaviviruses and the other of the Pestiviruses and Hepaciviruses. The latter two genera are known to be evolutionarily distant from the Flaviviruses as reflected in the structure-based phylogeny [104,105]. The structural divergence of RdRps between the members of *Flaviviridae* is mostly because of the varied amino terminus associated with the “in cis” regulation of the core polymerase [24,30]. It, in turn, leads to the different disposition of the motifs G and F (residues 90–100 and residues 135–160, respectively, in HCV and their equivalents in others) resulting in significant structural deviations [24,30]. Apart from the conserved structural elements around the catalytic center, the Hepaci and Pestiviruses share significant similarity near the carboxy-terminal region due to the presence of a 21-residue membrane anchor [104].

Members of *Birnaviridae* [22,45] are the only ds RNA viruses that use protein priming for initiating replication, as evidenced by the conserved VPg sequence motif at the N-terminus of their RdRps. The members of *Permutotetraviridae* such as *Thosea asigna virus* (TAV) [106] also share this feature. Additionally, the RdRps of *Birnaviridae and Permutotetraviridae* permute cyclically due to the non-canonical C-A-B arrangement of the motifs instead of the regular A-B-C leading to an altered active site geometry [22,45,106]. However, the conserved GDD motif is absent in Birnaviruses (present in TAV) making them catalytically less efficient. Further, their fingertips do not interact with the thumb subdomain. These features indicate a separate lineage for *Birnaviridae* that is distinct from other dsRNA viruses as apparent in the phylogenetic profile (Figure 3C).

Unlike Birnaviral RdRps, the RdRp of hPBV from *Picobirnaviridae* has the characteristic GDD motif and the canonical arrangement of A-B-C motifs [46]. It, on the one hand, bears structural resemblances to Caliciviral RdRps [20] in possessing a wide active site cavity and on the other hand, to Flaviviral RdRps in possessing a distinctive long loop that serves as the “primer grip” [24,30]. The presence of the long insertion loop in the thumb subdomain of hPBV RdRp [46] is possibly the main reason for its placement in the same sub-clade along with HCV and BVDV (Figure 3C). The RdRp of TAV seems to share key structural features with Birnaviruses, Picobirnaviruses as well as the Flaviviruses, therefore, justifying its position in the phylogenetic tree.

The *Leviviridae* group [28,95] is considered phylogenetically distant from the *Cystoviridae* [42,44], and much uncertainty still exists regarding the origin of both the groups. Nevertheless, the bacterial phages originate from the same ancestral node indicating conservation of the core structural elements that are suitable for the more straightforward semi-conservative mode of transcription, which eventually paved the way for the wholly conservative method of transcription seen in *Reoviridae* [29]. Thus, the polymerases of *Reoviridae* have evolved to possess a four-channeled architecture unlike the three-channeled ones for the RdRps employing semi-conservative RNA synthesis [29,69,71]. It is probably for this reason that *Cystoviridae*, *Flaviviridae*, *Picobirnaviridae*, and *Birnaviridae* share the same sub-clade, which is distinct from *Reoviridae* in the structural dendrogram (Figure 3C).

Understandably, the RdRps of *Orthomyxoviridae* and *Bunyaviridae* share structural features of *Reoviridae* in terms of using a capped RNA oligonucleotide for initiation of transcription, and for a combination of terminal and internal de novo initiation of genome replication [56,62]. Further, their RdRps also have four channels like those of *Reoviridae*. Hence, they are grouped under the same sub-clade (Figure 3C). The unique feature of the ss (−) RNA viruses include the presence of an exclusive template exit channel, the absence of conserved G in the GDD motif, and the association of structural motif G with the PA subunit in Influenza viruses and PA-C like domain in LACV instead of the core polymerase region, to mention a few [49,50,51,62]. While the origin of segmented ss (−) viruses is proposed to be from *Flaviviridae*, there is much uncertainty regarding the origin of *Mononegavirales* [100,101,102]. The ss (−) RNA viruses are a recently evolved group as they have limited host range comprising of plants and animals. The horizontal transfer of viruses between plant and animal hosts via arthropod and nematode vectors is proposed to be the primary contributor to the evolution of the *Mononegavirales* [100,101,102].

The RdRps of Group IV viruses, *Caliciviridae* infecting animals [38,39,40,41] and *Picornaviridae* [23,70,84,85,86,87,88,89,90] infecting vertebrates are structurally very similar in having a wide active site to facilitate protein-mediated initiation of replication using VPg [19,20]. Naturally, they are part of the clade comprising viruses originating from the picorna-like superfamily (Figure 3C) [100,101,102]. Unfortunately, this clade lacks the structural presence of RdRps from other diverse eukaryotic viruses that share the same evolutionary lineage.

Overall, the structural phylogeny reflects the gross and finer modifications in RdRps that have occurred during evolution to suit individual replication strategies of viruses from different groups.

## 4. Analysis of RdRp Complexes

As of September 2017, 486 structures of RdRps and their complexes were deposited at the PDB. Of these, a set of 260 ligand-bound structures, determined to a resolution better than 2.5 Å, were retrieved. About 200 unique ligands were selected from this set and analyzed for understanding the interactions and associations with RdRps (Table 2). The most common ligands associated with the RdRps include the divalent metal ions such as magnesium, manganese, calcium, and zinc. The divalent metal ions are crucial for polymerization reaction as they coordinate the catalytic aspartates and facilitate the formation of a phosphodiester bond between NTPs [6,71]. Of the two metal ions, one is permanently bound to the protein, whereas the other is weakly associated and gets stabilized at different stages of catalysis [107]. The metal ions help in positioning the NTP’s triphosphate group optimally for attack by the sugar moiety of the nascent strand. It is highly likely that the absence of divalent ions in the crystal structures of some RdRps might be due to their loss during purification [108]. Studies in *Flaviviridae* and *Cystoviridae* show that the bound noncatalytic ions facilitate initiation of replication and the transition to elongation stages by promoting the displacement of the C-terminal domain [108]. Apart from these, ions such as phosphate, chloride, sulfate, and pyrophosphate, moieties such as glycerol, propionic and acetic acid that are most probably constituents of crystallization buffers are also observed regularly in crystal structure complexes. Structural details of 121 RdRp complexes with inhibitors were retrieved from the list of unique ligands while only 20 different complexes were present of RdRps bound to substrates and their derivatives (Table 2). 

### 4.1. RdRp Complexes in Reoviridae

**Figure 4:**
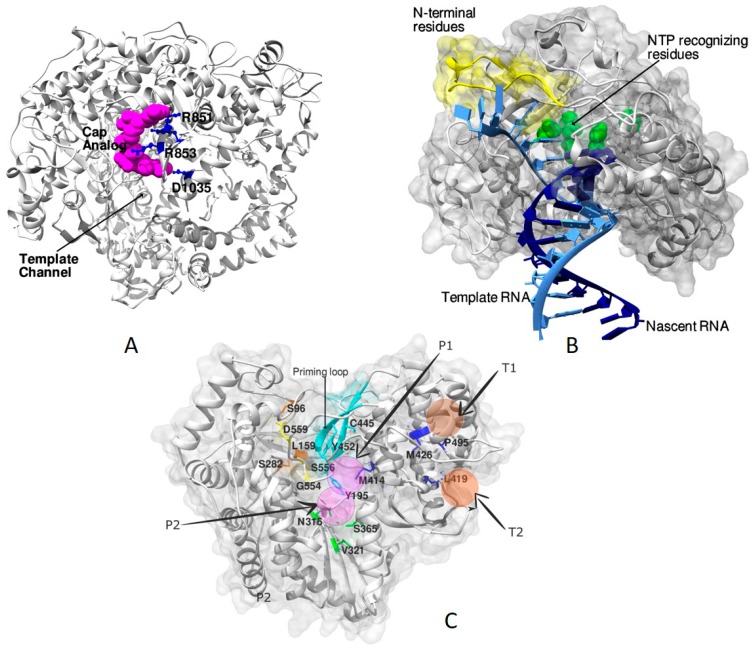
Structures of RdRp complexes of different viruses: (**A**) Figure showing the cap-binding site of MRV3 (PDB ID: 1mwh) [29] on the surface of λ3 polymerase. The cap-analog is shown as spheres of magenta color, and the interacting residues are depicted in ball-and-stick representation in blue color. The binding of cap helps in distinguishing the (+) RNA from the (−) RNA and facilitates transcription [29]. (**B**) The course of entry of template RNA (light blue) and the exit of dsRNA (template and nascent RNA) after polymerization as observed in the PV RdRp (PDB ID: 3ol6) [23]. The residues of the amino terminus (shown in yellow) are demonstrated to have a significant influence on both catalysis and fidelity during RNA synthesis [23]. Residues D233, D238, S288, G289, N297, and D328 that take part in NTP recognition and catalysis are shown as green color spheres. (**C**) Potential allosteric sites are seen in the HCV RdRp associated with the thumb and the palm subdomains. The four sites are depicted as transparent spheres in the figure; two each belonging to the thumb and the palm subdomains. The residues involved in conferring resistance to nucleoside analogs (yellow) and other NNIs are indicated in stick representation. The priming loop is colored in cyan.

Detailed studies with the λ3 polymerase of MRV3 in complex with the cap analog, (7-methyl-guanosine-5’-triphosphate-5′ guanosine), RNA oligonucleotides, rNTPs and 3’-deoxy-NTP provide valuable insights into the mode of initiation, elongation, and encapsidation of dsRNA [29]. The study enabled the identification of the cap-binding site on the surface of the λ3 polymerase between the template channel and the RNA exit channel (Figure 4A). The methylated Guanosine of the cap-analog formed hydrogen bonds with Arg851 and Asp1035 of the C-terminal domain. In *Reoviridae*, the capping of the (+) strand of RNA helps in distinguishing it from the (−) strand and engaging the RdRp close to the 3′ end of the (−) strand to facilitate transcription [29,109]. A unique priming loop of the palm subdomain (residues 558–565) that supports the priming rNTPs acts as a kinetic barrier for the transition from initiation to elongation stages offering itself as a potential antiviral target [29]. The structure of elongation complexes indicated the spatial separation of the exit of nascent RNAs: one mode of exit via the front channel during replication and the other through the bottom during transcription to facilitate capping [29].

### 4.2. RdRp Complexes in Bacteriophages

X-ray crystal structures of the RdRps of Qβ and φ6 in complex with template RNA, NTPs, and their analogs are available. The structures suggest the molecular basis for de novo initiation, elongation and consequences of calcium binding [83,95]. The structure of RNA oligonucleotides that mimicked the conserved 3′ end of the (−) strand of the genome with the φ6 polymerase revealed the plasticity of binding and interactions of RNA template with the residues in the template channel [110]. The nucleotides that are exterior to the template channel were seen to interact with the charged surface around the entrance driving the transport of template RNA through the tunnel. There were strong resemblances in the template positions of the bacteriophage φ6 initiation complexes with those of the previously described reoviral λ3 initiation complexes [29]. Further, the basis of calcium-mediated inhibition was rationalized using the crystal structure in which the altered positioning of one of the calcium ions prevented catalysis by distorting the geometry of the active site. The study suggests the pivotal role of the concentration of divalent metal ions both inside and outside the polymerase in controlling its activity [83].

In bacteriophage Qβ, the crystal structure of the core replicase in complex with a template RNA ending with CCA-3′ and complementary 7- or 8-mer RNA oligonucleotides are available [95]. In many Group IV viruses including bacteriophage Qβ, the CCA initiation boxes are seen to have the independent ability to direct transcription [111]. In the structure of the RdRp from Qβ, the adenosine of CCA-3′ served as a platform for the establishment of an initiation complex during de novo initiation while the penultimate C served as the first template nucleotide for polymerization. The β-subunit and EF-Tu interact with the backbone of the template and nascent RNAs, thereby guiding them towards the active site and exit channels, respectively. Unlike the calcium-mediated inhibition observed in the φ6 RdRp [83], there was normal catalysis with bound calcium in the Qβ complex [95]. It is due to the positioning of the calcium ions at sites equivalent to those of bound Mg^2+^ of elongation complexes of φ6 RdRp [83].

### 4.3. RdRp Complexes of Caliciviridae

Many RNA/NTP bound structures are available for the Group IV viruses. In Calicicviruses, the structures of RdRp complexes of hNoV reveal exciting approaches for inhibitor design using nucleotide analogs [112,113,114]. In these structures, binding of such analogs led to rearrangements at the catalytic site resulting in loss of activity. In the polymerase complex of hNoV with the inhibitor 5-nitrocytidine triphosphate, catalysis is blocked by the alteration of the substrate binding site due to the necessity of accommodating the nitro group of the inhibitor. This led to a closed conformation of the RdRp representing a trapped state immediately before nucleotidyl transfer reaction [114]. The complex of hNoV RdRp with the substrate analog 2’-amino-2’-deoxycytidine-5’-triphosphate shows rearrangement of the catalytic site and disruption of the coordination shells of the active-site metal ions due to the 2′ substitutions of the nucleotide [114]. The structure of a backtracked state of hNoV with substrate and primer complex, revealed a hybrid state where the conformation of the thumb subdomain was open while the active site was closed [113]. In the hybrid state, the central helix of the thumb subdomain rotated by 22° due to the movement of the C-terminal region away from the active-site cavity. These structures helped in elucidating the conformational changes associated with RNA translocation post catalysis, especially involving the central helix and the carboxy-terminal end, suggesting newer targets for the design of antivirals [20].

### 4.4. RdRp Complexes of Picornaviridae

A thorough study of the mode of NTP binding, elongation and catalysis has come from the structures of picornaviral RdRp complexes [115]. The structure of the complex of 3D^pol^ with GTP (PDB ID: 1ra7) provided deeper insights into the mechanism of proteolysis-dependent activation of the polymerase [23,116]. Though the polymerase was crystallized with different NTPs, the density for GTP was the most prominent owing to its superior Kd values (4 μM) over others. Similarly, a conspicuous density was observed for the GTP in the RdRp complexes of EVD68 [90,117]. Further, the binding of the N-terminus to a pocket at the back of the fingers subdomain significantly impacted the positioning of Asp 238 of the fingers subdomain by stabilizing a backbone structure that directly interacted with it. This activation was hypothesized to facilitate the movement of the NTP into the catalytic site for base pairing with the template and poising it for the phosphoryl transfer reaction [90,117]. Similar observations were made with PV elongation complexes where the residues of the amino terminus were shown to influence catalysis and fidelity aspects [19,23,116]. Studies involving the complexes of all four NTPs with 3Dpol revealed a common pre-insertion site to which the NTPs bind before catalysis. The interactions of the ring finger with the NTPs helped it to ratchet back and forth and drive the NTPs into the catalytic site [23]. Inhibitor complexes of *Picornaviridae* reveal new regions close to the template binding site at the interface of thumb and fingers that are suitable for the development of antivirals [19]. Structural studies on complexes of primer-template RNA with RdRps of *Foot-and-mouth disease virus* (FMDV) [87,118], PV (Figure 4B) [23,85,115,116], Coxsackievirus B3 (CVB3) [70,115], Human Rhinovirus 16 (HRV) [86,115], Enterovirus A71 (EV71) [89] and Enterovirus D68 (EVD68) [90,117] have enriched our understanding of molecular mechanisms of catalysis, particularly the dynamics in the palm subdomain associated with elongation. A six-state reference model was proposed based on a study involving PV elongation complexes (Figure 4B) [23] and is discussed by Peng Gong’s group in the same issue. The states S1 through S6 indicate series of changes in and around the active site that takes the polymerase from a catalytically open conformation ready for NTP binding in S1 to a closed state S3 necessitated by the requirement to achieve proper geometry for polymerization. The state S4 is attuned immediately following the catalysis and leads to the S5 state in which the polymerase reverts to the open conformation. The last state (S6) is proposed to be a hypothetical translocation intermediate state between S5 and S1 states for the next cycle of nucleotide addition that moves the RdRp one position downstream on its template. The crystal structure of EV71 RdRp elongation complexes with natural NTP substrate combinations and controlled incubation time helped in identifying the asymmetric movement of the template–product duplex during translocation [89]. These structures ascertained the fact that the aspects of fidelity and nucleotide selection in RdRps resulted from the accurate recognition of the Watson–Crick base pair geometry [89]. Additionally, the role of long-distance interactions was evident in setting the stage for rapid polymerization [19].

### 4.5. RdRp Complexes of Flaviviridae

Due to the severe health concerns associated with both HCV and DENV, the structural studies on RdRp complexes of Flaviviruses, particularly HCV, have surpassed those from any other group or individual virus. Pegylated interferons and ribavirin were routinely used for the treatment of HCV infection before 2013. However, the treatment is now replaced by Direct Acting Antivirals (DAAs) [119]. Among the DAAs of HCV RdRp, sofosbuvir and dasabuvir are approved for treatment while beclabuvir is currently in phase III clinical trials [119,120]. The potential DAAs include nucleoside analogs that are like the natural substrate, are phosphorylated before binding and lead to chain termination upon incorporation. The non-nucleoside inhibitors (NNIs) bind to regions other than the active site and inhibit enzyme activity [120,121,122]. Of the 114 unique structures of RdRp complexes of HCV with resolution better than 2.5 Å, only five are determined with the substrate and its analogs while the rest are NNI complexes comprising 109 unique ligands (Table 3).

An exhaustive study involving complexes of RdRp of HCV with RNA templates, primers, NTPs, nucleoside analogs and catalytic metal ions have provided useful insights into the role of the priming loop of the thumb subdomain and the C-terminal membrane-anchoring linker during de novo initiation and elongation [123]. Together, the thumb and the C-terminal linker act as “swinging gate” occluding the active-site cavity in the apo state, retracting during de novo and primed initiation and vacating the same during elongation. Complexes of HCV NS5b with the clinically active form of the drug sofosbuvir led to the identification of critical interactions that are interrupted upon drug binding, thereby locking the enzyme in its inactive apo form [124]. Studies with sofosbuvir and other nucleoside inhibitors have shown the importance of substitutions at S282, L159, S282, L320, and V321 in conferring resistance to the drugs (Figure 4C) [120].

The NNIs of HCV and DENV affect polymerase activity by interfering with crucial conformational changes associated with catalysis and elongation by preventing the contacts between the thumb and the fingers subdomain [75,122]. Five allosteric sites of HCV NS5b were identified (Figure 4C) including two in the thumb subdomain, two within the palm subdomain and one adjacent to the β-hairpin extending from the thumb subdomain (P-β) [120,122]. Palm subdomain inhibitors are proposed to bind to two pockets (P1 and P2) in the proximity of the active site [120,122]. About 55 palm site inhibitors have been reported and studied in detail (Table 3 and Table S1). These include derivatives of NNIs such as pyridazinones, cyclopentapyridines, quinolizinones, dialkylnaphthalenones, proline derivatives, acrylic acids, anthranilic acids, oxyfluorobenzamides, piperazine-2-Carboxamides, benzofurans, and benzodiazepines to mention a few [81,120]. There are 44 thumb subdomain inhibitors complexes (Table 3 and Table S1). These include derivatives of benzimidazoles and indoles, pyrazolopyrimidines, quinoxalines, and pyrazolylmethylacrylic acids, aurones, thiophene-2-Carboxylic acids, pyranoindoles, thiazolones, phenylalanine [82,125]. These inhibitors bind either at the junction between the thumb and the fingers subdomain (T1) or on the outer surface of the thumb subdomain (T2) [81,125]. The P-β inhibitors include derivatives of imidazopyridines identified from the metabolite tegobuvir that interact with the β hairpin extending from the thumb subdomain (Table 3 and Table S1) [120]. Several substitution mutants involving the residues of finger and palm subdomains, the β hairpin, and the C-terminal linker are identified to confer resistance to the NNIs (Figure 4C) [120].

Five PDBs represent structures of complexes of NNIs from the Novartis’ fragment collection of DENV RdRp obtained by X-ray crystallography [126]. The inhibitor based on biphenyl acetic acid fragment three was observed to bind to a new pocket in the palm subdomain with an IC_50_ of ≈700 µM. In a cell-based assay, another lead candidate that has a replacement of the carboxylic acid moiety with an isosteric acylsulfonamide displayed antiviral activity at low micromolar concentrations against all four DENV serotypes [126]. Many of the NNIs of HCV and DENV are in various phases of clinical trials and hold promise as potential drugs for the treatment of dengue and HCV infections.

## 5. Conclusions

The structures of monomeric RdRps from different viruses that are available in the PDB provide useful insights regarding the conservation of the core structural elements required for the functioning of this versatile molecule. Although the sequences have diverged significantly, the catalytic elements including the sequence motifs (DX_2-4_D and GDD) and the seven other structural motifs (A–G) are found to be conserved. The core structural elements of RdRps include those of DNA and RNA polymerases formed by the fingers, palm and thumb subdomains. Presence of additional domains and association with host proteins aid the RdRps in various processes associated with RNA synthesis. The structural comparisons of viruses from different groups indicate how the conformational elements have evolved to meet the requirements arising out of a change in genome or dependence on a primer. Analysis of ligand complexes provides deeper insights into the role of metal ions and the function of structural elements in template recognition and binding. The study of inhibitor complexes helps to take stock of the structural data of potential drug candidates that are currently available and understand their modes of inhibition.

## Figures and Tables

**Table 1:** Classification of RNA dependent RNA polymerases (RdRps) of different RNA viruses based on PROSITE accession no. PRU00539 [14].

S. No.	Category	PROSITE ID	Total Reviewed Sequences/Exemplar UniProt Accession No.	No. of Unique structures/Exemplar Protein Data Bank (PDB) IDs
1	Bacteriophages (Group III and IV)	PS50522	07	03
	*Leviviridae:*			
	*Bacteriophage Qβ* (Qβ)		P14647	3mmp (2.5 Å)
	*Cystoviridae:*			
	*Pseudomonas phage φ6* (φ6)		P11124	1hhs (2.0 Å)
	*Pseudomonas phage φ12* (φ12)		Q94M06	4gzk (1.69 Å)
2	*Reoviridae* (Group III)	PS50523	45	03
	Mammalian orthoreovirus 3 (MRV3)		P0CK31	1n35 (2.5 Å)
	Simian rotavirus SA11 (SiRV)		O37061	2r7r (2.6 Å)
	*Bombyx mori* Cytoplasmic polyhedrosis virus (BmCPV)		A0A0S1LIW6	5h0r (3.9 Å)
3	*Birnaviridae* (Group III)	PS50524	09	02
	*Infectious bursal disease virus* (IBDV)		Q9Q6Q5	2pus (2.4 Å)
	*Infectious pancreatic necrosis virus* (IPNV)		P22173	2yi9 (2.2 Å)
4	*Picobirnaviridae* (Group III)		01	01
	*Picobirnaviridae*			
	*Human picobirnavirus* (hPBV)		Q50LE4	5i61 (2.4 Å)
5	Group IV viruses	PS50507	470	18
	*Permutetraviridae*:			
	*Thosea asigna virus* (TAV)		Q6A562	4xhi (2.15 Å)
	*Picornaviridae:*			
	Coxsackievirus B3 (CVB3)		Q5UEA2	4zpc (1.59 Å)
	Human rhinovirus 16 (HRV)		Q82122	1xr7 (2.3 Å)
	Poliovirus type 1 (PV)		P03300	1ra6 (2.0 Å)
	*Foot-and-mouth disease virus* (FMDV)		Q9QCE4	1u09 (1.91 Å)
	Encephalomyocarditis virus 1 (EMCV)		P12296	4nyz (2.15 Å)
	Enterovirus A71 (EV71)		E5RPG2	5f8n (2.48 Å)
	Enterovirus D68 (EVD68)		F1T146	5xe0 (2.3 Å)
	*Caliciviridae:*			
	Murine Norovirus (mNoV)		Q80J95	3uqs (2.0 Å)
	Human Norovirus (hNoV)		A0ZNP5	4nrt (2.02 Å)
	*Sapporo virus* (SV)		Q69014	2uut (2.4 Å)
	*Rabbit hemorrhagic disease virus* (RHDV)		P27410	1khw (2.7 Å)
	*Flaviviridae:*			
	*Hepatitis C virus* (HCV)		O92972	1nb4 (2.0 Å)
	*Bovine viral diarrhea virus* (BVDV)		Q96662	2cjq (2.6 Å)
	*West nile virus* (WNV)		P14335	2hcn (2.35 Å)
	*Dengue virus* (DENV)		Q6YMS4	4hhj (1.79 Å)
	*Zika virus* (ZIKV)		A0A109PRQ3	5wz3 (1.8 Å)
	*Japanese encephalitis virus* (JEV)		P27395	4k6m (2.6 Å)
6	Group V viruses (segmented genome)	PS50525	142	04
	*Orthomyxoviridae:*			
	*Influenza A virus* (FluA)		H6QM91	5m3h (2.5 Å)
	*Influenza B virus* (FluB)		Q5V8Y6	4wrt (2.7 Å)
	*Influenza C virus* (FluC)		Q9IMP4	5d98 (3.9 Å)
	*Bunyaviridae:*			
	La crosse virus (LACV)		A5HC98	5amq (3.0 Å)
7	Group V viruses (non-segmented genomes)	PS50526	81	01
	*Rhabdoviridae:*			
	*Vesicular stomatitis virus* (VSV)		P03523	5a22 (3.8 Å)

**Table 2:** Inhibitor and substrate complexes of RdRPs from different viruses (listed in Table 1) determined to a resolution better than 2.5 Å.

Group	Family	Structures with Ligands	RdRps Complexed with nucleoside triphosphate (NTP) and Their Derivatives	PDB IDs of RdRp-NTP Complexes	RdRps Complexed with Inhibitors
dsRNA viruses	*Reoviridae* and *Birnaviridae*	5	2	1MWH,1N35	0
Bacteriophages	*Cystoviridae* and *Leviviridae*	9	2	3AVX, 1UVK	0
(+) ssRNA Viruses	*Caliciviridae*	6	4	3B5N, 3BS0, 3H5X, 3H5Y	1
(+) ssRNA Viruses	*Flaviviridae*	133	5	2XI3, 1GX6, 1GX5, 4HDH, 4HDG	120
(+) ssRNA Viruses	*Picornaviridae*	40	7	1RA7, 2ILZ, 2IM0, 2IM1, 2IM2, 3OLB, 5F8I	0
(−) ssRNA Viruses	*Ortho-myxoviridae*, *Rhabdoviridae*	5	0	NA	0

**Table 3:** List of the structures of non-nucleoside inhibitor complexes of the RdRp of HCV.

Binding Sites	PDB IDs	Total No. of Complexes
Palm Site Inhibitor Complexes	3CDE, 3CWJ, 2YOJ, 3BR9, 3BSA, 3CO9, 3CVK, 3D28, 3D5M, 3E51, 3G86, 3GYN, 3H2L, 3H59, 3H5S, 3H5U, 3H98, 3HKW, 3HKY, 3LKH, 3SKA, 3SKE, 3SKH, 3TYQ, 3TYV, 3U4O, 3U4R, 3UPH, 3UPI, 4EAW, 4IH5, 4IH6, 4IH7, 4KAI, 4KB7, 4KBI, 4KE5, 4KHM, 4KHR, 4MIB, 4MK8, 4MK9, 4MKA, 4MKB, 4MZ4, 5PZK, 5PZL, 5PZN, 3FQK, 3FQL, 4JY0, 2GIQ, 2AWZ, 2AX0, 2AX1	55
Thumb Site Inhibitor Complexes	2D3U, 2D3Z, 2D41, 2HWH, 2HWI, 1NHU, 2DXS, 2O5D, 2WHO, 3CIZ, 3CJ0, 3CJ2, 3CJ3, 3CJ4, 3CJ5, 3FRZ, 3MF5, 3Q0Z, 4DRU, 4EO6, 4EO8, 4IZ0, 4J02, 4J04, 4J06, 4J08, 4J0A, 4JJS, 4JJU, 4JU3, 4JU4, 4JU6, 4JU7, 4JVQ, 4OBC, 4TLR, 2BRK, 2BRL, 2HAI, 2I1R, 2WRM, 3HVO, 2WCX, 2GIR	44
Primer Grip Inhibitor Complex	2IJN, 5TWM,1YVF	3
At interfaces of Subdomains	2GC8, 3GNW, 3QGF, 3QGH, 3QGI, 5TRI, 5TRK	7

## Supplementary Materials

The following are available online at http://www.mdpi.com/1999-4915/10/2/76/s1. Table S1: List of the structures of non-nucleoside inhibitor complexes of the RdRp of HCV.
