# Supplementary material for: RNA Dependent RNA Polymerases: Insights from Structure, Function and Evolution
Source: Viruses. 2018 Feb 10;10(2):76. doi: 10.3390/v10020076 (PMC5850383; doi:10.3390/v10020076)
Supplement: Supplementary file 1 [file viruses-10-00076-s001.docx]

Supplementary Table S1: List of the structures of non-nucleoside inhibitor complexes of the RNA dependent RNA polymerase of Hepatitis C Virus.

| Binding Sites | PDB IDs | Formula | Inhibitor name |
| --- | --- | --- | --- |
| Palm Site (55 Complexes) | 3CDE | C_21_ H_23_ N_5_ O_6_ S_3_ | N-{3-[5-hydroxy-2-(3-methylbutyl)-3-oxo-6-thiophen-2-yl-2,3-dihydropyridazin-4-yl]-1,1-dioxido-2H-1,2,4-benzothiadiazin-7-yl}methanesulfonamide |
|  | 3CWJ | C_22_ H_24_ N_4_ O_6_ S_3_ | N-{3-[5-hydroxy-2-(3-methylbutyl)-3-oxo-6- thiophen-2-yl-2,3-dihydropyridazin-4-yl]- 1,1-dioxido-2H-1,4-benzothiazin-7-yl}methanesulfonamide |
|  | 2YOJ | C_25_ H_14_ F_2_ N_4_ O_5_ | 4-fluoranyl-6-[(7-fluoranyl-4-oxidanylidene-3H-quinazolin-6-yl)methyl]-8-(2-oxidanylidene-1H-pyridin-3-yl)furo[2,3-e]indole-7-carboxylic acid |
|  | 3BR9 | C_23_ H_25_ N_5_ O_6_ S_2_ | (2R)-2-({3-[5-hydroxy-2-(3-methylbutyl)-3-oxo-6-thiophen-2-yl-2,3-dihydropyridazin-4-yl]-1,1-dioxido-2H-1,2,4-benzothiadiazin-7-yl}oxy)propanamide |
|  | 3BSA | C_21_ H_22_ N_6_ O_6_ S_2_ | 2-({3-[5-hydroxy-2-(3-methylbutyl)-3-oxo-6-(1,3-thiazol-5-yl)-2,3-dihydropyridazin-4-yl]-1,1-dioxido-2H-1,2,4-benzothiadiazin-7-yl}oxy)acetamide |
|  | 3CO9 | C_20_ H_23_ N_5_ O_6_ S_2_ | N-{3-[4-hydroxy-1-(3-methylbutyl)-2-oxo-1,2-dihydropyrrolo[1,2-b]pyridazin-3-yl]-1,1-dioxido-2H-1,2,4-benzothiadiazin-7-yl}methanesulfonamide |
|  | 3CVK | C_21_ H_29_ N_5_ O_6_ S_2_ | N-{3-[1-(3,3-Dimethyl-butyl)-4-hydroxy-2-oxo-1,2,4a,5,6,7-hexahydro-pyrrolo[1,2-b]pyridazin-3-yl]-1,1-dioxo-1,2-dihydro-1lambda6-benzo[1,2,4]thiadiazin-7-yl}-methanesulfonamide |
|  | 3D28 | C_21_ H_20_ N_2_ O_4_ S | (5S)-1-benzyl-3-(1,1-dioxido-1,2-benzisothiazol-3-yl)-4-hydroxy-5-(1-methylethyl)-1,5-dihydro-2H-pyrrol-2-one |
|  | 3D5M | C_24_ H_25_ C_l_ F N_3_ O_6_ S_2_ | N-({3-[(5S)-5-tert-butyl-1-(3-chloro-4-fluorobenzyl)-4-hydroxy-2-oxo-2,5-dihydro-1H-pyrrol-3-yl]-1,1-dioxido-1,2-benzisothiazol-7-yl}methyl)methanesulfonamide |
|  | 3E51 | C_21_ H_28_ N_6_ O_6_ S_2_ | N-{3-[5-hydroxy-2-(3-methylbutyl)-3-oxo-6-pyrrolidin-1-yl-2,3-dihydropyridazin-4-yl]-1,1-dioxido-2H-1,2,4-benzothiadiazin-7-yl}methanesulfonamide |
|  | 3G86 | C_25_ H_19_ F_2_ N_3_ O_6_ S_2_ | N-{3-[6-fluoro-1-(4-fluorobenzyl)-4-hydroxy-2-oxo-1,2-dihydroquinolin-3-yl]-1,1-dioxido-4H-1,4-benzothiazin-7-yl}methanesulfonamide |
|  | 3GYN | C_24_ H_34_ N_4_ O_6_ S_2_ | N-{3-[(5R)-1-cyclopentyl-4-hydroxy-5-methyl-5-(3-methylbutyl)-2-oxo-1,2,5,6-tetrahydropyridin-3-yl]-1,1-dioxido-4H-1,2,4-benzothiadiazin-7-yl}methanesulfonamide |
|  | 3H2L | C_23_ H_23_ F N_4_ O_6_ S_2_ | N-{3-[(4ar,7as)-1-(4-fluorobenzyl)-4-hydroxy-2-oxo-2,4a,5,6,7,7a-hexahydro-1H-cyclopenta[b]pyridin-3-yl]-1,1-dioxido-2H-1,2,4-benzothiadiazin-7-yl}methanesulfonamide |
|  | 3H59 | C_26_ H_30_ F N_3_ O_6_ S_2_ | N-{3-[(5S)-5-(1,1-dimethylpropyl)-1-(4-fluoro-3-methylbenzyl)-4-hydroxy-2-oxo-2,5-dihydro-1H-pyrrol-3-yl]-1,1-dioxido-4H-1,4-benzothiazin-7-yl}methanesulfonamide |
|  | 3H5S | C_27_ H_30_ F N_3_ O_6_ S_2_ | (5S)-5-tert-butyl-1-(4-fluoro-3-methylbenzyl)-4-hydroxy-3-[8-(methylsulfonyl)-1,1-dioxido-6,7,8,9-tetrahydroisothiazolo[4,5-h]isoquinolin-3-yl]-1,5-dihydro-2H-pyrrol-2-one |
|  | 3H5U | C_24_ H_26_ F N_3_ O_6_ S_2_ | N-({3-[(5S)-5-tert-butyl-1-(4-fluorobenzyl)-4-hydroxy-2-oxo-2,5-dihydro-1H-pyrrol-3-yl]-1,1-dioxido-1,2-benzisothiazol-7-yl}methyl)methanesulfonamide |
|  | 3H98 | C_19_ H_22_ N_6_ O_6_ S_2_ | N-{3-[5-hydroxy-8-(3-methylbutyl)-7-oxo-7,8-dihydroimidazo[1,2-a]pyrimidin-6-yl]-1,1-dioxido-4H-1,2,4-benzothiadiazin-7-yl}methanesulfonamide |
|  | 3HKW | C_30_ H_32_ F N_3_ O_6_ S | (11S)-10-[(2,5-dimethyl-1,3-oxazol-4-yl)carbonyl]-11-{2-fluoro-4-[(2-methylprop-2-en-1-yl)oxy]phenyl}-3,3-dimethyl-2,3,4,5,10,11-hexahydrothiopyrano[3,2-b][1,5]benzodiazepin-6-ol 1,1-dioxide |
|  | 3HKY | C_30_ H_32_ F N_3_ O_6_ S | (11S)-10-[(2,5-dimethyl-1,3-oxazol-4-yl)carbonyl]-11-{2-fluoro-4-[(2-methylprop-2-en-1-yl)oxy]phenyl}-3,3-dimethyl-2,3,4,5,10,11-hexahydrothiopyrano[3,2-b][1,5]benzodiazepin-6-ol 1,1-dioxide |
|  | 3LKH | C_23_ H_29_ F N_2_ O_4_ | 2-(2-{[(1S)-1-benzyl-2-hydroxyethyl]amino}-2-oxoethoxy)-N-butyl-6-fluoro-N-methylbenzamide |
|  | 3SKA | C_21_ H_15_ F_3_ N_4_ O_3_ | 1-[(2-aminopyridin-4-yl)methyl]-3-(2-oxo-1,2-dihydropyridin-3-yl)-5-(trifluoromethyl)-1H-indole-2-carboxylic acid |
|  | 3SKE | C_22_ H_14_ F_3_ N_5_ O_4_ S | 1-[(2-aminopyridin-4-yl)methyl]-3-(2,4-dioxo-1,2-dihydrothieno[3,4-d]pyrimidin-3(4H)-yl)-5-(trifluoromethyl)-1H-indole-2-carboxylic acid |
|  | 3SKH | C_22_ H_15_ C_l_ F N O_2_ | 1-benzyl-5-chloro-3-(2-fluorophenyl)-1H-indole-2-carboxylic acid |
|  | 3TYQ | C_23_ H_18_ F N_3_ O_5_ | 5-ethyl-1-(2-fluoro-5-nitrobenzyl)-3-(2-oxo-1,2-dihydropyridin-3-yl)-1H-indole-2-carboxylic acid |
|  | 3TYV | C_25_ H_20_ F_3_ N_3_ O_4_ S | N-(cyclopropylsulfonyl)-1-(2,5-difluorobenzyl)-6-fluoro-5-methyl-3-(2-oxo-1,2-dihydropyridin-3-yl)-1H-indole-2-carboxamide |
|  | 3U4O | C_20_ H_15_ C_l_ N_4_ O_3_ | 1-[(2-aminopyridin-4-yl)methyl]-5-chloro-3-(2-oxo-1,2-dihydropyridin-3-yl)-1H-indole-2-carboxylic acid |
|  | 3U4R | C_27_ H_23_ C_l_ N_6_ O_6_ S_2_ | 1-[(2-aminopyridin-4-yl)methyl]-5-chloro-N-({3-[(methylsulfonyl)amino]phenyl}sulfonyl)-3-(2-oxo-1,2-dihydropyridin-3-yl)-1H-indole-2-carboxamide |
|  | 3UPH | C_24_ H_19_ F_2_ N_3_ O_5_ S | 6-(2,5-difluorobenzyl)-N-(methylsulfonyl)-8-(2-oxo-1,2-dihydropyridin-3-yl)-3,6-dihydro-2H-furo[2,3-e]indole-7-carboxamide |
|  | 3UPI | C_25_ H_21_ F_2_ N_3_ O_5_ S | (3S)-6-(2,5-difluorobenzyl)-3-methyl-N-(methylsulfonyl)-8-(2-oxo-1,2-dihydropyridin-3-yl)-3,6-dihydro-2H-furo[2,3-e]indole-7-carboxamide |
|  | 4EAW | C_24_ H_37_ N_4_ O_6_ P S | N-{(1S)-3-[(5S)-5-tert-butyl-1-(3,3-dimethylbutyl)-4-hydroxy-2-oxo-2,5-dihydro-1H-pyrrol-3-yl]-1-ethoxy-1-oxido-1,4-dihydro-2,4,1-benzodiazaphosphinin-7-yl}methanesulfonamide |
|  | 4IH5 | C_11_ H_10_ N_6_ | 4-(2-phenylhydrazinyl)-1H-pyrazolo[3,4-d]pyrimidine |
|  | 4IH6 | C_17_ H_24_ N_2_ O_2_ | (5S)-3-(4-tert-butylbenzyl)-5-(propan-2-yl)imidazolidine-2,4-dione |
|  | 4IH7 | C_15_ H_17_ N O | 3-(3-tert-butylphenyl)pyridin-2(1H)-one |
|  | 4KAI | C_27_ H_25_ B F_2_ N_2_ O_6_ S | [4-({[5-cyclopropyl-2-(4-fluorophenyl)-3-(methylcarbamoyl)-1-benzofuran-6-yl](methylsulfonyl)amino}methyl)-2-fluorophenyl]boronic acid |
|  | 4KB7 | C_29_ H_28_ B F N_2_ O_6_ S | 5-cyclopropyl-2-(4-fluorophenyl)-6-[{2-[(3R)-1-hydroxy-1,3-dihydro-2,1-benzoxaborol-3-yl]ethyl}(methylsulfonyl)amino]-N-methyl-1-benzofuran-3-carboxamide |
|  | 4KBI | C_28_ H_25_ B F_2_ N_2_ O_6_ S | 5-cyclopropyl-6-{[(7-fluoro-1-hydroxy-1,3-dihydro-2,1-benzoxaborol-5-yl)methyl](methylsulfonyl)amino}-2-(4-fluorophenyl)-N-methyl-1-benzofuran-3-carboxamide |
|  | 4KE5 | C_27_ H_25_ B F_2_ N_2_ O_6_ S | [4-({[5-cyclopropyl-2-(4-fluorophenyl)-3-(methylcarbamoyl)-1-benzofuran-6-yl](methylsulfonyl)amino}methyl)-2-fluorophenyl]boronic acid |
|  | 4KHM | C_27_ H_25_ B F_2_ N_2_ O_6_ S | [4-({[5-cyclopropyl-2-(4-fluorophenyl)-3-(methylcarbamoyl)-1-benzofuran-6-yl](methylsulfonyl)amino}methyl)-2-fluorophenyl]boronic acid |
|  | 4KHR | C_27_ H_25_ B F_2_ N_2_ O_6_ S | [4-({[5-cyclopropyl-2-(4-fluorophenyl)-3-(methylcarbamoyl)-1-benzofuran-6-yl](methylsulfonyl)amino}methyl)-2-fluorophenyl]boronic acid |
|  | 4MIB | C_25_ H_32_ N_4_ O_4_ S | N-({(3S)-1-[6-tert-butyl-5-methoxy-8-(2-oxo-1,2-dihydropyridin-3-yl)quinolin-3-yl]pyrrolidin-3-yl}methyl)methanesulfonamide |
|  | 4MK8 | C_25_ H_30_ N_2_ O_4_ S | N-(4-{2-[3-tert-butyl-2-methoxy-5-(2-oxo-1,2-dihydropyridin-3-yl)phenyl]ethyl}phenyl)methanesulfonamide |
|  | 4MK9 | C_24_ H_25_ N_3_ O_5_ S | N-{2-[3-tert-butyl-2-methoxy-5-(2-oxo-1,2-dihydropyridin-3-yl)phenyl]-1,3-benzoxazol-5-yl}methanesulfonamide |
|  | 4MKA | C_26_ H_26_ N_2_ O_6_ S | N-{3-[3-tert-butyl-2-methoxy-5-(2-oxo-1,2-dihydropyridin-3-yl)phenyl]-1-oxo-1H-isochromen-7-yl}methanesulfonamide |
|  | 4MKB | C_24_ H_27_ N_3_ O_4_ S | N-(4-{(E)-2-[3-tert-butyl-2-methoxy-5-(3-oxo-2,3-dihydropyridazin-4-yl)phenyl]ethenyl}phenyl)methanesulfonamide |
|  | 4MZ4 | C_25_ H_17_ C_l_ F N_3_ O_3_ | 1-[(2-chloroquinolin-3-yl)methyl]-6-fluoro-5-methyl-3-(2-oxo-1,2-dihydropyridin-3-yl)-1H-indole-2-carboxylic acid |
|  | 5PZK | C_38_ H_38_ N_4_ O_5_ | (2E)-3-(4-{[(1-{[(13-cyclohexyl-6-oxo-6,7-dihydro-5H-indolo[1,2-d][1,4]benzodiazepin-10-yl)carbonyl]amino}cyclopentyl)carbonyl]amino}phenyl)prop-2-enoic acid |
|  | 5PZL | C_23_ H_21_ F N_4_ O_6_ S | 2-({3-[1-(2-cyclopropylethyl)-6-fluoro-4-hydroxy-2-oxo-1,2-dihydroquinolin-3-yl]-1,1-dioxo-1,4-dihydro-1lambda~6~,2,4-benzothiadiazin-7-yl}oxy)acetamide |
|  |  | C_23_ H_21_ F N_4_ O_6_ S | 2-({3-[1-(2-cyclopropylethyl)-6-fluoro-4-hydroxy-2-oxo-1,2-dihydroquinolin-3-yl]-1,1-dioxo-1,4-dihydro-1lambda~6~,2,4-benzothiadiazin-7-yl}oxy)acetamide |
|  | 5PZN | C_27_ H_25_ F N_2_ O_3_ | 5-[3-(tert-butylcarbamoyl)phenyl]-2-(4-fluorophenyl)-N-methyl-1-benzofuran-3-carboxamide |
|  | 5PZN | C_38_ H_38_ N_4_ O_5_ | (2E)-3-(4-{[(1-{[(13-cyclohexyl-6-oxo-6,7-dihydro-5H-indolo[1,2-d][1,4]benzodiazepin-10-yl)carbonyl]amino}cyclopentyl)carbonyl]amino}phenyl)prop-2-enoic acid |
|  | 3FQK | C_22_ H_23_ F N_2_ O_5_ S | 5-cyclopropyl-2-(4-fluorophenyl)-6-[(2-hydroxyethyl)(methylsulfonyl)amino]-N-methyl-1-benzofuran-3-carboxamide |
|  | 3FQL | C_22_ H_23_ F N_2_ O_5_ S | 5-cyclopropyl-2-(4-fluorophenyl)-6-[(2-hydroxyethyl)(methylsulfonyl)amino]-N-methyl-1-benzofuran-3-carboxamide |
|  | 4JY0 | C_27_ H_32_ N_4_ O_3_ S | (4S,5R)-1-(4-tert-butylbenzoyl)-2-(2-methylpropyl)-4-pyrazin-2-yl-5-(1,3-thiazol-2-yl)-L-proline |
|  | 2GIQ | C_21_ H_18_ F N_3_ O_4_ S | 1-(2-cyclopropylethyl)-3-(1,1-dioxido-2h-1,2,4-benzothiadiazin-3-yl)-6-fluoro-4-hydroxyquinolin-2(1h)-one |
|  | 2AWZ | C_16_ H_13_ Br N_2_ O_3_ S_3_ | 5r-(4-bromophenylmethyl)-3-(benzenesulfonylamino)-4-oxo-2-thionothiazolidine |
|  | 2AX0 | C_19_ H_18_ N_2_ O_3_ S_3_ | 5r-(2e-methyl-3-phenyl-allyl)-3-(benzenesulfonylamino)-4-oxo-2-thionothiazolidine |
|  | 2AX1 | C_14_ H_10_ C_l2_ N_2_ O_3_ S_4_ | 5r-(3,4-dichlorophenylmethyl)-3-(2-thiophenesulfonylamino)-4-oxo-2-thionothiazolidine |
| Thumb Site  (44 Complexes) | 2D3U | C_19_ H_14_ N_2_ O_4_ S_2_ | 5-(4-cyanophenyl)-3-{[(2-methylphenyl)sulfonyl]amino}thiophene-2-carboxylic acid |
|  | 2D3Z | C_18_ H_14_ F N O_4_ S_2_ | 5-(4-fluorophenyl)-3-{[(4-methylphenyl)sulfonyl]amino}thiophene-2-carboxylic acid |
|  | 2D41 | C_19_ H_17_ N O_5_ S_3_ | 5'-acetyl-4-{[(2,4-dimethylphenyl)sulfonyl]amino}-2,2'-bithiophene-5-carboxylic acid |
|  | 2HWH | C_16_ H_14_ N_2_ O_4_ S_2_ | 4-methyl-n-{(5e)-5-[(5-methyl-2-furyl)methylene]-4-oxo-4,5-dihydro-1,3-thiazol-2-yl}benzenesulfonamide |
|  | 2HWI | C_18_ H_15_ F N_2_ O_4_ S | (2s)-({(5z)-5-[(5-ethyl-2-furyl)methylene]-4-oxo-4,5-dihydro-1,3-thiazol-2-yl}amino)(4-fluorophenyl)acetic acid |
|  | 1NHU | C_24_ H_18_ C_l2_ F_3_ N O_3_ | (2s)-2-[(2,4-dichloro-benzoyl)-(3-trifluoromethyl-benzyl)-amino]-3-phenyl-propionic acid |
|  | 2DXS | C_27_ H_30_ N_2_ O_4_ | N-[(13-cyclohexyl-6,7-dihydroindolo[1,2-d][1,4]benzoxazepin-10-yl)carbonyl]-2-methyl-l-alanine |
|  | 2O5D | C_24_ H_19_ F N_4_ O_7_ S_2_ | (2s)-2-({(5z)-5-[(5-ethyl-2-furyl)methylene]-4-oxo-4,5-dihydro-1,3-thiazol-2-yl}amino)-2-(4-fluorophenyl)-n-[(4-nitrophenyl)sulfonyl]acetamide |
|  | 2WHO | C_20_ H_15_ BR N_2_ O3 | 2-(3-bromophenyl)-6-[(2-hydroxyethyl)amino]-1h-benzo[de]isoquinoline-1,3(2h)-dione |
|  | 3CIZ | C_7_ H_6_ Br N O_2_ | 2-amino-5-bromobenzoic acid |
|  | 3CJ0 | C_9_ H_9_ Br N_2_ O_3_ | 4-[(5-bromopyridin-2-yl)amino]-4-oxobutanoic acid |
|  | 3CJ2 | C_14_ H_19_ Br N_2_ O | 4-bromo-2-{[(3R,5S)-3,5-dimethylpiperidin-1-yl]carbonyl}aniline |
|  | 3CJ3 | C_18_ H_18_ Br C_l_ N_2_ O | 4-bromo-2-{[(2R)-2-(2-chlorobenzyl)pyrrolidin-1-yl]carbonyl}aniline |
|  | 3CJ4 | C_18_ H_23_ Br N_2_ O_4_ | 4-[(4-bromo-2-{[(3R,5S)-3,5-dimethylpiperidin-1-yl]carbonyl}phenyl)amino]-4-oxobutanoic acid |
|  | 3CJ5 | C_22_ H_30_ Br N_3_ O_4_ | N-(4-bromo-2-{[(3R,5S)-3,5-dimethylpiperidin-1-yl]carbonyl}phenyl)-4-morpholin-4-yl-4-oxobutanamide |
|  | 3FRZ | C_29_ H_37_ N_5_ O_3_ | (6R)-6-cyclopentyl-6-[2-(2,6-diethylpyridin-4-yl)ethyl]-3-[(5,7-dimethyl[1,2,4]triazolo[1,5-a]pyrimidin-2-yl)methyl]-4-hydroxy-5,6-dihydro-2H-pyran-2-one |
|  | 3FRZ | C_35_ H_39_ N_3_ O_7_ | N-[(benzyloxy)carbonyl]-L-alpha-glutamyl-N-[(1S)-4-oxo-4-phenyl-1-propylbut-2-en-1-yl]-L-phenylalaninamide |
|  | 3MF5 | C_24_ H_24_ O_2_ S | 3-[2-(trans-4-methylcyclohexyl)phenyl]-5-phenylthiophene-2-carboxylic acid |
|  | 3Q0Z | C_38_ H_38_ N_4_ O_5_ | (2E)-3-(4-{[(1-{[(13-cyclohexyl-6-oxo-6,7-dihydro-5H-indolo[1,2-d][1,4]benzodiazepin-10-yl)carbonyl]amino}cyclopentyl)carbonyl]amino}phenyl)prop-2-enoic acid |
|  | 4DRU | C_32_ H_38_ N_4_ O_5_ S | 13-cyclohexyl-3-methoxy-17,22-dimethyl-7H-10,6-(methanoiminothioiminobutanoiminomethano)indolo[2,1-a][2]benzazepine-14,23-dione 16,16-dioxide |
|  | 4EO6 | C_22_ H_31_ N O_3_ S | 5-(3,3-dimethylbut-1-yn-1-yl)-3-{[(trans-4-methylcyclohexyl)carbonyl](propan-2-yl)amino}thiophene-2-carboxylic acid |
|  | 4EO8 | C_21_ H_30_ N_2_ O_3_ S | 5-(3,3-dimethylbut-1-yn-1-yl)-3-{2,2-dimethyl-1-[(trans-4-methylcyclohexyl)carbonyl]hydrazinyl}thiophene-2-carboxylic acid |
|  | 4IZ0 | C_10_ H_7_ C_l3_ N_2_ O_3_ S | 2,4,5-trichloro-N-(5-methyl-1,2-oxazol-3-yl)benzenesulfonamide |
|  | 4J02 | C_16_ H_17_ C_l2_ N O_3_ | [(1R)-5,8-dichloro-1-propyl-1,3,4,9-tetrahydropyrano[3,4-b]indol-1-yl]acetic acid |
|  | 4J04 | C_13_ H_7_ C_l4_ N O_4_ S | 4-chloro-2-{[(2,4,5-trichlorophenyl)sulfonyl]amino}benzoic acid |
|  | 4J06 | C_11_ H_7_ Br C_l_ N O_4_ S_2_ | 2-{[(5-bromothiophen-2-yl)sulfonyl]amino}-4-chlorobenzoic acid |
|  | 4J08 | C_20_ H_17_ N O_5_ S | 2-{[(4-methylphenyl)sulfonyl]amino}-5-phenoxybenzoic acid |
|  | 4J0A | C_20_ H_17_ N O_5_ S | 2-{[(4-methylphenyl)sulfonyl]amino}-4-phenoxybenzoic acid |
|  | 4JJS | C_25_ H_28_ F_3_ N O_4_ | 2-{[(trans-4-methylcyclohexyl)carbonyl](propan-2-yl)amino}-5-[2-(trifluoromethyl)phenoxy]benzoic acid |
|  | 4JJU | C_21_ H_12_ F_5_ N_3_ O_2_ | 1-(2,4-difluorobenzyl)-6-{[3-(trifluoromethyl)pyridin-2-yl]oxy}quinazolin-4(1H)-one |
|  | 4JU3 | C_21_ H_17_ N O_7_ S | 5-(4-carboxyphenoxy)-2-{[(4-methylphenyl)sulfonyl]amino}benzoic acid |
|  | 4JU4 | C_19_ H_13_ Br F N O_5_ S | 2-{[(4-bromo-2-fluorophenyl)sulfonyl]amino}-5-phenoxybenzoic acid |
|  | 4JU6 | C_24_ H_29_ N O_4_ | 2-{[(trans-4-methylcyclohexyl)carbonyl](propan-2-yl)amino}-5-phenoxybenzoic acid |
|  | 4JU7 | C_24_ H_29_ N O_4_ | 2-{[(trans-4-methylcyclohexyl)carbonyl](propan-2-yl)amino}-5-phenoxybenzoic acid |
|  | 4JVQ | C_32_ H_36_ N_2_ O_6_ | 5-{4-[(4-methoxybenzoyl)amino]phenoxy}-2-{[(trans-4-methylcyclohexyl)carbonyl](propan-2-yl)amino}benzoic acid |
|  | 4OBC | C_6_ H_13_ N O_4_ S | 2-(n-morpholino)-ethanesulfonic acid |
|  |  | C_12_ H_26_ O_6_ | 1-(2-methoxy-ethoxy)-2-{2-[2-(2-methoxy-ethoxy]-ethoxy}-ethane |
|  | 4TLR | C_24_ H_33_ N O_5_ S | 3-{(2R,5R)-5-cyclohexyl-2-[(2R)-2-hydroxypropyl]-3-oxomorpholin-4-yl}-5-(3,3-dimethylbut-1-yn-1-yl)thiophene-2-carboxylic acid |
|  |  | C_22_ H_23_ F N_2_ O_5_ S | 5-cyclopropyl-2-(4-fluorophenyl)-6-[(2-hydroxyethyl)(methylsulfonyl)amino]-N-methyl-1-benzofuran-3-carboxamide |
|  | 2BRK | C_27_ H_30_ N_2_ O_4_ | 3-cyclohexyl-1-(2-morpholin-4-yl-2-oxoethyl)-2-phenyl-1h-indole-6-carboxylic acid |
|  | 2BRL | C_31_ H_39_ N_3_ O_3_ | 3-cyclohexyl-1-(2-{methyl[(1-methylpiperidin-3-yl)methyl]amino}-2-oxoethyl)-2-phenyl-1h-indole-6-carboxylic acid |
|  | 2HAI | C_5_ H_11_ N O_3_ S_2_ | S,s-(2-hydroxyethyl)thiocysteine |
|  |  | C_3_ H_7_ N O_4_ S | 3-sulfinoalanine |
|  |  | C_21_ H_27_ F O_4_ | (6s)-6-cyclopentyl-6-[2-(3-fluoro-4-isopropoxyphenyl)ethyl]-4-hydroxy-5,6-dihydro-2h-pyran-2-one |
|  | 2I1R | C_18_ H_15_ F N_6_ O_2_ S | (5z)-5-[(5-ethyl-2-furyl)methylene]-2-{[(s)-(4-fluorophenyl)(1h-tetrazol-5-yl)methyl]amino}-1,3-thiazol-4(5h)-one |
|  | 2WRM | C_18_ H_18_ N_2_ O_4_ | (3r)-3-(4-methyl-1,3-dioxo-1,3-dihydro-2h-pyrrolo[3,4-c]quinolin-2-yl)hexanoic acid |
|  | 3HVO | C_20_ H_15_ BR N_2_ O_3_ | 2-(3-bromophenyl)-6-[(2-hydroxyethyl)amino]-1h-benzo[de]isoquinoline-1,3(2h)-dione |
|  | 2WCX | C_20_ H_21_ N O_2_ S | 6-cyclohexyl-4-methyl-5-phenyl-4h-thieno[3,2-b]pyrrole-2-carboxylic acid |
|  | 2GIR | C_22_ H_27_ N O_3_ S | 3-{isopropyl[(trans-4-methylcyclohexyl)carbonyl]amino}-5-phenylthiophene-2-carboxylic acid |
| Primer Grip (3 Complexes) | 2IJN | C_12_ H_9_ F_6_ N_3_ O S | (2r,3r)-3-{[3,5-bis(trifluoromethyl)phenyl]amino}-2-cyano-3-thioxopropanamide |
|  | 5TWM | C_28_ H_29_ F N_4_ O_3_ | 5-[3-(tert-butylcarbamoyl)phenyl]-6-(ethylamino)-2-(4-fluorophenyl)-N-methylfuro[2,3-b]pyridine-3-carboxamide |
|  | 1YVF | C_22_ H_16_ Br N O_4_ | (2z)-2-(benzoylamino)-3-[4-(2-bromophenoxy)phenyl]-2-propenoic acid |
| Subdomains interfaces  (7 Complexes) | 2GC8 | C_12_ H_15_ C_l_ N_2_ O_4_ S | 1-[(2-amino-4-chloro-5-methylphenyl)sulfonyl]-l-proline |
|  | 3GNW | C_34_ H_32_ F N_3_ O_5_ S | (11S)-11-[4-(benzyloxy)-2-fluorophenyl]-3,3-dimethyl-10-[(6-methylpyridin-2-yl)carbonyl]-2,3,4,5,10,11-hexahydrothiopyrano[3,2-b][1,5]benzodiazepin-6-ol 1,1-dioxide |
|  | 3QGF | C_38_ H_38_ N_4_ O_5_ | (2E)-3-(4-{[(1-{[(13-cyclohexyl-6-oxo-6,7-dihydro-5H-indolo[1,2-d][1,4]benzodiazepin-10-yl)carbonyl]amino}cyclopentyl)carbonyl]amino}phenyl)prop-2-enoic acid |
|  |  | C_24_ H_23_ C_l_ F_3_ N_5_ O_5_ S | (2R)-4-(6-chloropyridazin-3-yl)-N-(4-methoxybenzyl)-1-{[4-(trifluoromethoxy)phenyl]sulfonyl}piperazine-2-carboxamide |
|  | 3QGH | C_28_ H_26_ F_6_ N_6_ O_6_ S | N-cyclopropyl-6-[(3R)-3-{[4-(trifluoromethoxy)benzyl]carbamoyl}-4-{[4-(trifluoromethoxy)phenyl]sulfonyl}piperazin-1-yl]pyridazine-3-carboxamide |
|  | 3QGI | C_29_ H_30_ F_6_ N_6_ O_6_ S | N-[(2S)-butan-2-yl]-6-[(3R)-3-{[4-(trifluoromethoxy)benzyl]carbamoyl}-4-{[4-(trifluoromethoxy)phenyl]sulfonyl}piperazin-1-yl]pyridazine-3-carboxamide |
|  | 5TRI | C_22_ H_16_ C_l_ N O_4_ | 3-[(4-chlorophenyl)methoxy]-2-(1-oxo-1,3-dihydro-2H-isoindol-2-yl)benzoic acid |
|  |  | C3_8_ H_38_ N_4_ O_5_ | (2E)-3-(4-{[(1-{[(13-cyclohexyl-6-oxo-6,7-dihydro-5H-indolo[1,2-d][1,4]benzodiazepin-10-yl)carbonyl]amino}cyclopentyl)carbonyl]amino}phenyl)prop-2-enoic acid |
|  | 5TRK | C_23_ H_19_ C_l_ N_2_ O_5_ | N-{3-[(benzenecarbonyl)amino]-4-[(4-chlorophenyl)methoxy]benzene-1-carbonyl}glycine |
|  |  | C_38_ H_38_ N_4_ O_5_ | (2E)-3-(4-{[(1-{[(13-cyclohexyl-6-oxo-6,7-dihydro-5H-indolo[1,2-d][1,4]benzodiazepin-10-yl)carbonyl]amino}cyclopentyl)carbonyl]amino}phenyl)prop-2-enoic acid |
